# Validation of the recycled backfill material for the landslide stabilization at a railway line

**DOI:** 10.1038/s41598-024-57555-4

**Published:** 2024-03-25

**Authors:** Karmen Fifer Bizjak, Barbara Likar

**Affiliations:** Department of Geotechnics and Traffic Infrastructure, ZAG, Slovenian National Building and Civil Engineering Institute, Ljubljana, Slovenia

**Keywords:** Landslides, Recycled backfill material, Paper sludge ash, Paper sludge, Geotechnical composite, Railway line, Recycled material, Environment, Environmental sciences, Energy science and technology

## Abstract

In mountain areas landslides many times endanger safety of transport infrastructures, and these must be stabilized with retaining wall structures. In this paper the validation of a new composite as a backfill material for landslide stabilization with a large scale demo retaining wall is presented. The new composite was made from residues of paper industry, which uses for its production deinking process. New composite was validated with the laboratory tests, construction of small demo sites and at the end with a large demo retaining wall structure with a length of 50 m. It was concluded that the paper sludge ash and the paper sludge are in proportion 70:30, compacted on the optimal water content and maximum dry density, reached sufficient uniaxial compressive and shear strength. However, the composite's hydration processes required the definition of an optimal time between the composite mixing and installation. In 2019, the retaining wall structure from the new composite was successfully built. The large demo structure is an example of the knowledge transfer from the laboratory to the construction site, in which composite and installing technology could be verified.

## Introduction

In mountain areas landslides affect safety of transport infrastructures and represent a threat to roads and railways^1–3^. Retaining wall structures, which come in different types and shapes, are most often used to stabilize landslides. Analyses of landslides near railways showed that 70% of them are caused by human activities^4^. Rehabilitation costs are high, especially if transport congestion costs are considered^5^.

Twenty percent of the extracted materials are used for runways, railways, and waterways. These data vary across Europe due to differences in building tradition, natural resources, climate, and the state of the economy. In spite of the large quantities, reuse and recycling only amounts to 10.6%. The last Waste Framework Directive^6^ states that construction and demolition waste have to be re-used, recycled, or backfilled. These include the re-entry of construction and demolition of remove wastes, excavated materials, industrial wastes and marine sediments into the production cycle and the reuse of existing foundations^7^. This directive envisaged an increased use of recycled materials from other industries that can be used as backfill material.

Due to digitalisation, the worldwide production of graphic paper decreases, but the overall paper industry grows due to the production of packaging paper and hygiene and textile paper products^8^. In 2018, global paper production hit 400 million Mg per year^9^ and the vision of the paper industry is to maximize the content of recycled fibre. With the increase in paper production, landfills are necessary for such high production. Most paper residues are used in energy production by burning in boilers^10^. Paper ash, which is a residue remaining after burning, is a non-hazardous waste material, but landfilling with this material will be reduced due to EU waste management legislative measures and policies^11^. Large landfills are required for such high quantities of ash and because of this it is difficult to obtain suitable locations.

The composition of ashes and sludge varies significantly from one paper mill to another^12^. It is, thus, necessary to conduct detailed investigations into the use of paper residues for geotechnical structures. Recent geotechnical research using paper sludge ash has been focused on optimisation processes for soil stabilization, using the sludge as a binder in road structures and embankments or as a backfill material in open pit mines^13^. Most of the published research was performed in the laboratory on previously prepared mixtures^14,15^. Paper sludge ash was used for soft soil stabilization, and laboratory tests were conducted with different percentages of paper sludge ash (5 to 20%). In these experiments, the uniaxial strength of the soil doubled after a 10% addition of ash^16,17^. A clay soil with high water content was stabilised with paper sludge ash, and the measurements showed that paper sludge ash can be used without any other binding additives^18^. In another experiment, several mixtures consisting of an expansive soil and paper sludge ash as a binder were obtained^19^. An optimum of 8% paper sludge ash showed an improvement in compressive strength. A clayey soil below a pavement was treated with paper sludge ash due to a very low bearing capacity. The results showed that paper sludge ash increased the strength of the soil and improved other geotechnical parameters such as the Atterberg limit, California bearing Ratio, and compaction ratio^20^. A paper sludge ash mixture with lime was compared with Portland cement and other additives for soil and road stabilization^21^. Also sulphate-bearing soil was stabilised with paper sludge ash instead of Portland cement and lime. Laboratory prepared samples showed that the linear expansion was significantly reduced with this mixture^22^.

Paper fly ash could completely replace the cement component in backfill material^23^. A 10% paper fly ash mixture reached compressive strengths of up to 0.8 MPa, which is high enough for geotechnical structures and roads. The mechanical properties of a recycled aggregate concrete (RAC) could also be improved with paper sludge ash and chemical properties with the higher resistance against acid and sulphate attacks^24^.

Even though most of the published investigations focus on laboratory experiments, some results of field investigations are also available. For example, subgrade of road infrastructure was stabilised with cement and paper sludge on a length of 250 m. After one week, the uniaxial compressive strength reached 4.5 MPa^25^. Likewise, for hydraulically bound layers, fly ash from paper industry was used. Field measurements showed that the bearing capacity increasing with time and paper fly ash is a suitable additive for road stabilization^26^.

Hazardous trace elements could be present in fly ash^27^, which makes its use impossible in earth structures^28^ and fill material^29–31^. It is, thus, mandatory to carry out leaching tests to avoid a negative impact on the environment.

Investors, designers, and construction companies are very conservative about using recycled material due to a lack in experience with such materials. At the same time, legislation on this topic varies from country to country, which makes it difficult to use recycled material as a construction product. The here presented large demo structure from recycled material was built to prove the possibility of using this composite as a backfill material.

In this paper new composite was developed from the residues of the paper industry with the deinking process. The new composited was made by 70% of paper sludge ash (PSA) and 30% of paper sludge (PS). The new composite was validated by laboratory tests, small demo fields, to validate the compacting technology and finally with the large demo construction of the retaining wall. The retaining wall was built near a rail track to stabilize an active landslide.

## Materials and methods

### Geotechnical condition of the landslide

**Figure 1:**
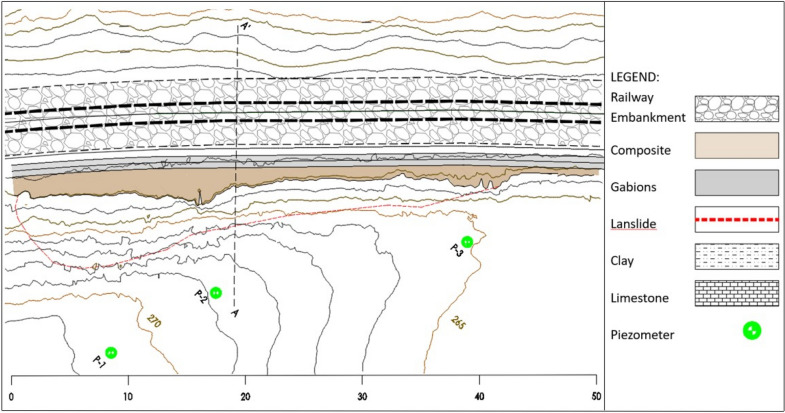
Location of the landslide.

**Figure 2:**
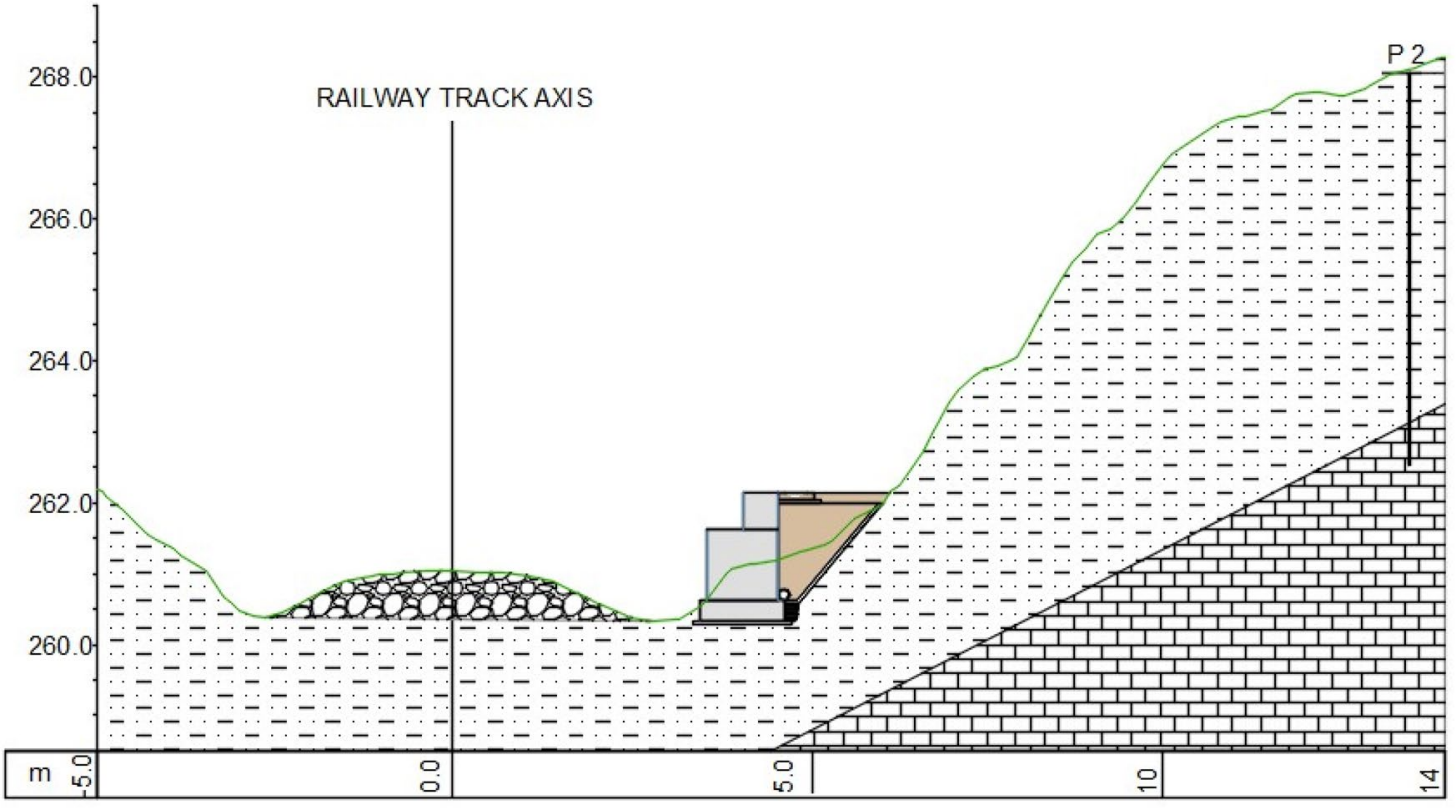
Geological profile A-A′

In mountainous countries, there is a high potential for landslides along transport lines. If the transport constructions are old and dilapidated, landslide vulnerability is even greater. In the southern part of Slovenia between Ljubljana and Novo mesto, an unstable slope endangered a very frequented railway line. Based on the geological investigation (Figs. 1, 2) of the landslide^32^, it was evident that a retaining wall structure had to be installed to prevent the landslide from further movements.

Firstly, a geomechanical investigation of the landslide area (900 m^2^) was performed and based on the results the final location for the retaining wall was selected. A 50 m long structure was envisaged for the first part of the landslide (300 m^2^) stabilization. The investigation program consisted of the drilling of nine boreholes on a length of 5 to 15 m, Standard penetration tests, pressure meter tests, and geomechanical laboratory tests on samples taken from the boreholes. Basic geomechanical properties of the soil were tested: water content and density^33^, Atterberg limits^34^, direct shear tests^35^, oedometer test^36^, permeability^37^ and Pocket penetrometer test. The boreholes were equipped with piezometers and inclinometers for further monitoring of the water level and slope deformations. The boreholes are designed in such a way that it is possible to perform inclinometric and piezometric measurements.

### Waste material—paper sludge ash and paper sludge

Residues from the paper industry were used for the large demo retaining wall structure. The raw material for the production process was recovered paper after the deinking process. Waste material from the production is paper sludge (PS), which is incinerated in steam boilers. A combustion residue from this steam boiling is paper sludge ash (PSA). PSA and PS are classified as non-hazardous waste without environmentally harmful components.

Chemical composition of PSA and PS are presented in Table 1. Also, leachate tests were done and showed that only barium content (63 mg/kg d.s.) exceeds the limit value for inert waste (20 mg/kg d.s.).

**Table 1:** Chemical composition of the waste material.

Waste material	Parameter (wt%)										
	Si	Al	Fe	Ca	P	Mg	K	Na	Ti	S	LOI
PSA	5.31	4.37	0.27	19.20	0.07	1.02	0.22	0.13	0.11	0.12	27.26
PS	3.4	2.94	0.17	12.73	0.03	0.62	0.13	0.10	0.07	0.02	53.41

Physico-mechanical properties of PSA and PS were tested in an accredited geomechanical laboratory according to the EU standards for the geomechanical testing of soil material, including the analysis of the water content, Atterberg limits, density, Proctor tests, unconfined compressive strength, and sieving analyses.

### Experimental design

The production of aggregates amounts to 3.07 billion Mg per year and the aggregate industry is the largest extraction industry in Europe^38^. Problems with the mixing and compaction of different materials often occur at the construction site, which cannot be identified in the laboratory where the material is prepared under ideal conditions. Therefore, before building the retaining wall, small demo sites were established for testing the mixing and compaction technology.

**Figure 3:**
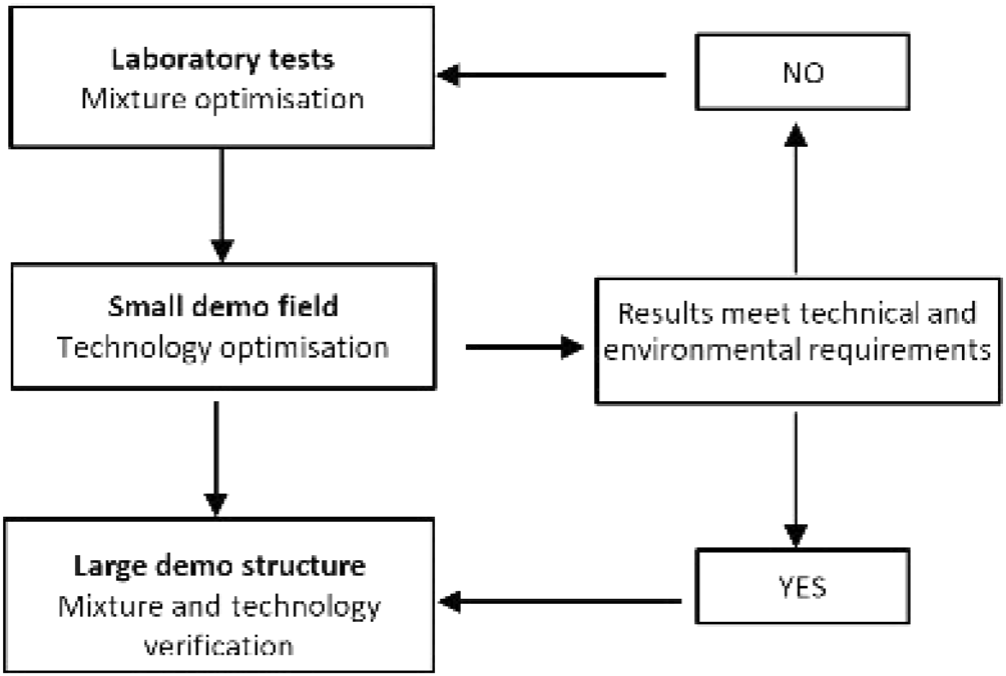
Experimental program.

The research on the new recycled material started with laboratory tests. These tests were performed on small samples, but at the construction site, the quantities of the used material were much larger. Therefore, it was necessary to build small test fields before installing new composites into large geotechnical structures. In this way, the mixing and compacting could be optimised. After technology optimization, the new composites could be successfully installed into the large demo structure, i.e. backfill material behind the retaining wall. The experimental procedure is shown in Fig. 3.

#### Laboratory tests

Geomechanical tests were performed in an accredited geomechanical laboratory. Mixtures with different content of PSA were tested but, in the paper, only results of composite with 70% PSA and 30% PS are presented. This mixture was chosen because it had sufficient geomechanical characteristics to be used as a backfill material and was, at the same time, suitable for compaction at the construction site.

The components of PSA and PS were mixed and then compacted at optimal water content to the maximum dry density. The Proctor Compaction Tests—SPP were done according to the requirements of the corresponding standard^39^ and additional compressive strength^40^. The tests were done with different time periods; directly after compaction and after one, four, seven, twenty-eight, and fifty days of curing. For direct shear tests^35^ some samples were tested immediately after compaction and others left to cure for 7 or 28 days before testing. Direct shear test was performed under the shear speed of 0.01 mm/min and vertical load of 100 kPa, 200 kPa and 400 kPa. The friction angle and cohesion were determined from the line that best fits test data by using the least squares method.

For freezing/thawing tests, the Slovenian technical specification^41^ TSC 06.320 (2001) was used. In a climate chamber, composites were freezing at − 20 °C and thawing at 20 °C at 12 cycles.

If material is not mixed at the construction site, the transport time is very important. The time delay between mixing and compaction was tested on samples which were moistened to the optimal water content and maximum water content (5% higher from the optimal water content). Two different procedures were used for the prepared samples. The mixture which was compacted at the maximum water content was cured in air (w_max_). The other material compacted at the optimum water content was cured in closed boxes (w_opt_).

#### Preparation of small demo fields and field testing

Small demo sites of 2 × 2 × 0,6 m in size were constructed with three layers, each of them having a thickness between 10 and 15 cm. PSA, PS, and water were mixed in a stirrer and then compacted to a density of at least 95% ρ_d,max_, as determined in the laboratory by the SPP.

The first demo site was compacted immediately after mixing, the next one 4 h after mixing, and the last one 24 h after mixing. For the last mixture, additional water was added, and remixing was needed for proper compaction.

Every installed layer was tested for dry density and moisture content (ρ_d,max_, w), according to^42^. The light weight deflectometer was used for determining the dynamic deformation modules (E_vd_)^43^ of every installed layer. For leaching tests according to^44^, the samples were taken from the demo sites after 28 days.

### Large demo retaining wall construction

#### Field and laboratory tests during demo retaining wall construction

**Figure 4:**
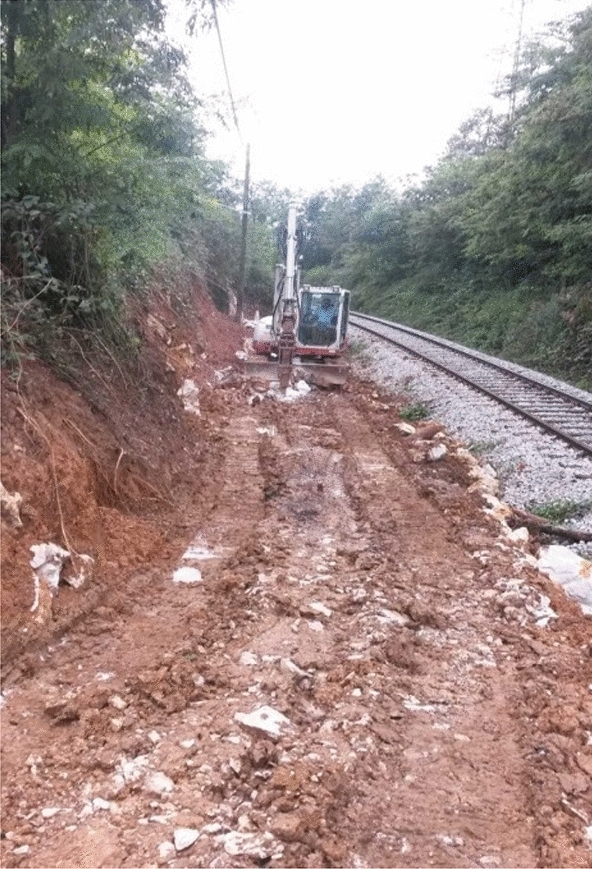
Location of the large demo structure.

**Figure 5:**
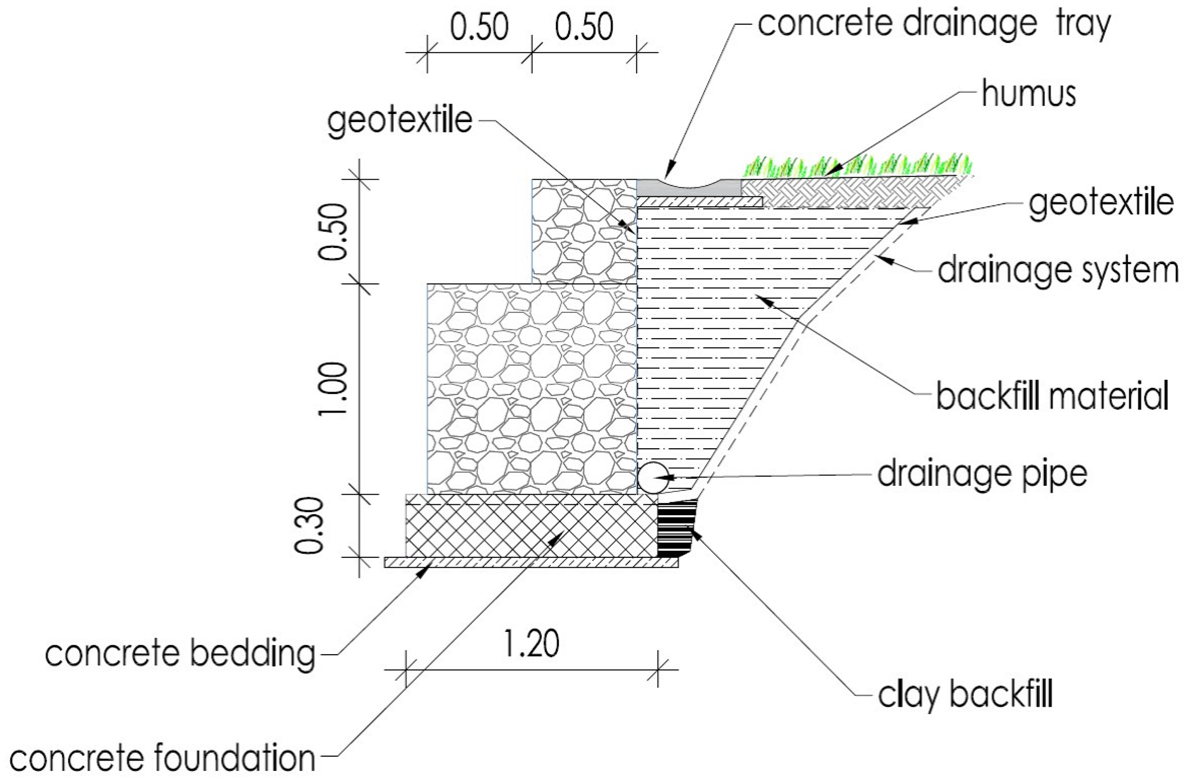
Profile of the retaining wall structure with backfill material.

**Figure 6:**
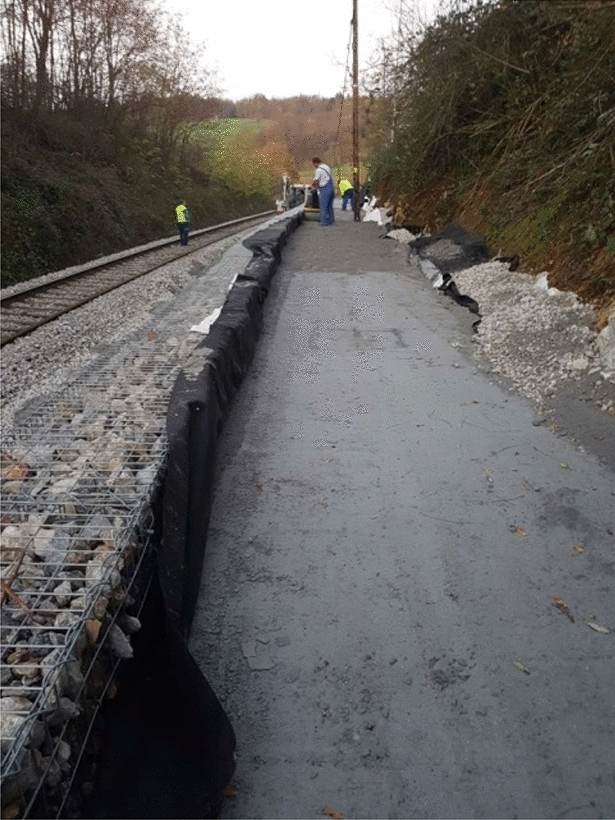
Compacted layer of the composite (8th layer).

Construction works started with earth work for the foundation of the 50 m long and 1,5 high retaining wall structure (Fig. 4) made by gabions in two lines. The foundation of the retaining wall structure was below the slip surface predicted from the borehole logging and geological mapping. The composite was installed between the landslide slope and gabions in a width between 2 and 3.5 m (Fig. 5). For the backfill material, 100 mg of mixture were used and compacted in nine layers. PSA and PS were mixed at the paper company facility, 70 km from the construction site. Based on the good results from the small test fields, we determined a thickness of 30 cm for the large demo construction (Fig. 6) and using hand operated mini road roller compactor. Thicker layers and a stronger compactor enabled faster construction of the large demo retaining wall. The water content and maximal dry density (w, ρ_d,max_) were measured by a nuclear surface gauges according to Slovenian technical specification. Moreover, dynamic deformation (E_vd_) modules were measured on each installed layer. Samples for direct shear and leachate tests were taken from the last layer. Samples for leachate tests were prepared according to SIST EN 1744-3 to clarify potential environmental impacts. Two samples were taken from the last layer and cured 28 days. In the installed material the temperatures are measured in different depths (0.30 m—T1, 0.60 m—T2 and T4 and 0.90 m—T3), while the water content is measured at depth of 0.60 m. The temperature probe (T4) was installed near the gabions while other three were installed in the middle point between gabions and natural slope.

#### Monitoring of the structure

Slope stability monitoring was performed during and after the construction phase via inclinometer measurements and a detailed laser scanning of the whole structure. A weather station was installed near the structure to register detailed climate conditions on the landslide.

The drainage system of the structure consists of vertical and horizontal pipes on the bottom of the composite and on the contact with the slope. A part of the water from the drainage system was collected in a plastic tank built at the end of the structure for water quality monitoring and chemical analyses to estimate environmental impacts on the surrounding areas.

## Results and discussion

### Geomechanical laboratory tests and field tests of the landslide material

Laboratory tests of samples from the landslide showed that the upper layers were mostly made of high plasticity clay, and partly of low plasticity clay. The lowest clay plasticity, compressibility modulus and shear properties were recorded at the contact between the rock and clay (Table 2) at a depth of 7–9 m in the highest part of the slope. Fissured dolomite was located below the clay layer.

**Table 2:** Geomechanical properties at the contact between the rock and clay.

Water content	Bulk density	Oedometer modulus at 200 kPa	w_p_	w_l_	I_p_	Permeability at 200 Pa	Friction angle/peak	Cohesion/peak
%	mg/m^2^	(MPa)	%	%	%	m/s	°	kPa
51	1.14	6	33	110	77	1E−9	10.5	32

Clay layers have a low permeability coefficient, therefore indicating that precipitation water mostly flows on the surface and on the contact between clay and limestone. Stability analyses of the landslide showed that the slip surface was^32^ at the contact of the clay and limestone in the depth of 9 m, which was confirmed with inclinometer measurements in the borehole above the rail line.

### Raw waste material

The initial water content of PS was 45–50%, while PSA was dry (Table 3). The optimum water content (*w*_*op*t_) determined with the standard Proctor test was higher and the maximum dry density (ρ_d,max_) lower for PS in comparison with the results for PSA. The unconfined compressive strength of PSA was like that of very stiff soil. For PS, the uniaxial compressive strength was much lower (220 kPa). Both materials were non-plastic. The results show that PSA was fined-grained, with an average particle size less than 63 µm.

**Table 3:** Properties of the raw material.

	Initial water content	Optimum water content	Maximum dry density	Unconfined compressive strength	Plastic limit	Particle size (mm)		
						0.063–2.5 mm	0.002–0.063	< 0.002
	%	%	mg/m^3^	(MPa)	%	%	%	%
PSA	0	51	0.99	0.3–0.5	Non plastic	13.3	75.59	11.11
PS	45–50	56.5	0.89	0.22	Non plastic	–	–	–

### Properties of the composite

In the laboratory several mixtures were prepared and among them the mixture C70/30 reached enough high geomechanical properties.

#### Standard proctor tests (SPP)

Standard Proctor tests for the mixture with 70% PSA and 30% PS (C70/30) indicated that the w_opt_ was between 45 and 50% and the ρ_d,max_ was between 0.99 and 1 mg/m^3^.

The main difference between the investigated composites and the natural gravel material was the density. The ρ_d,max_ of natural gravel material is between 1.98 and 2.12 mg/m^3^^45^, while the investigated composites had much lower ρ_d,max_ values. Also, mixture with 20% of fly ash and soil^46^ has higher ρ_d,max_ (1.48 mg/m^3^) than the composite C70/30. In the case that the soil beneath the foundation of the supporting structure is very soft and the heavy structure could cause large soil settlement, a lighter backfill could be an added advantage. . However, the composite requires a lot of water, indicating that great attention must be paid on the proper mixing of both materials.

#### Unconfined compressive strength—q_u_ and time behavior of the composite

**Figure 7:**
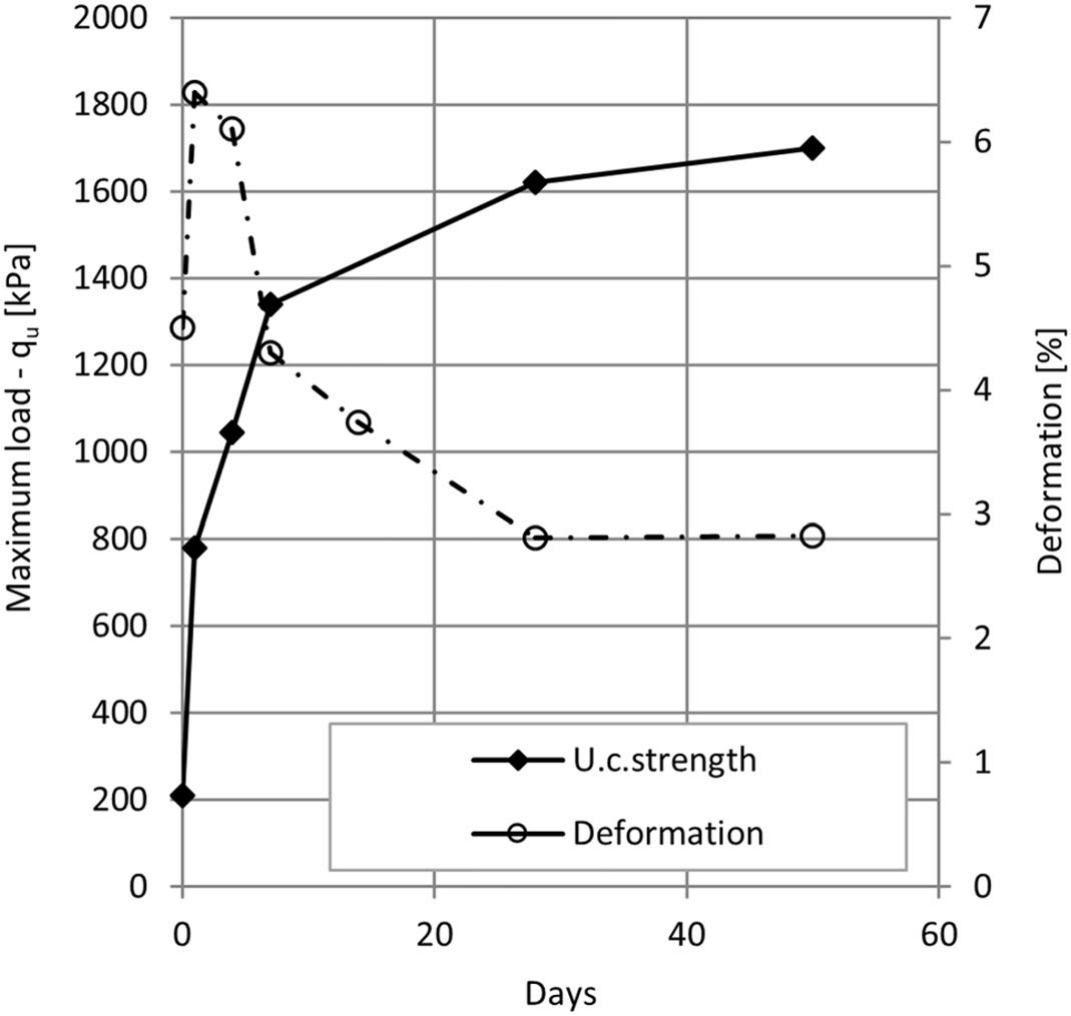
The vertical deformation at the maximum load after 0, 1, 4, 7, 28 and 50 days of curing.

Time of curing had an impact on the unconfined compressive strength of the composite and with time it increased. Immediately after mixing, *q*_*u*_ was low and in the range of soft soil, but after one week, *q*_*u*_ increased to over 1300 kPa (Fig. 7). After 28 days of curing, *q*_*u*_ was 1610 kPa and still slightly increased with time. Compared to the published data^23^, the mixture of sand, ash and a few % of sludge reached *q*_*u*_ of only 800 kPa after 28 days.

The time of curing had also a high impact on the vertical deformation at maximum load. A higher value of vertical deformation was observed after 1 day of curing, which rapidly decreased after 7 days. These points to the fact, that the time of curing had a high impact on the vertical deformation at maximum load. The behaviour of composites changed from ductile failure in the first 4 days to brittle in the next days of the sample’s observation. Even after the construction of the retaining wall, the creep subsides only after a certain time, so it is positive that the backfill material can be elastic and can withstand additional deformations.

**Figure 8:**
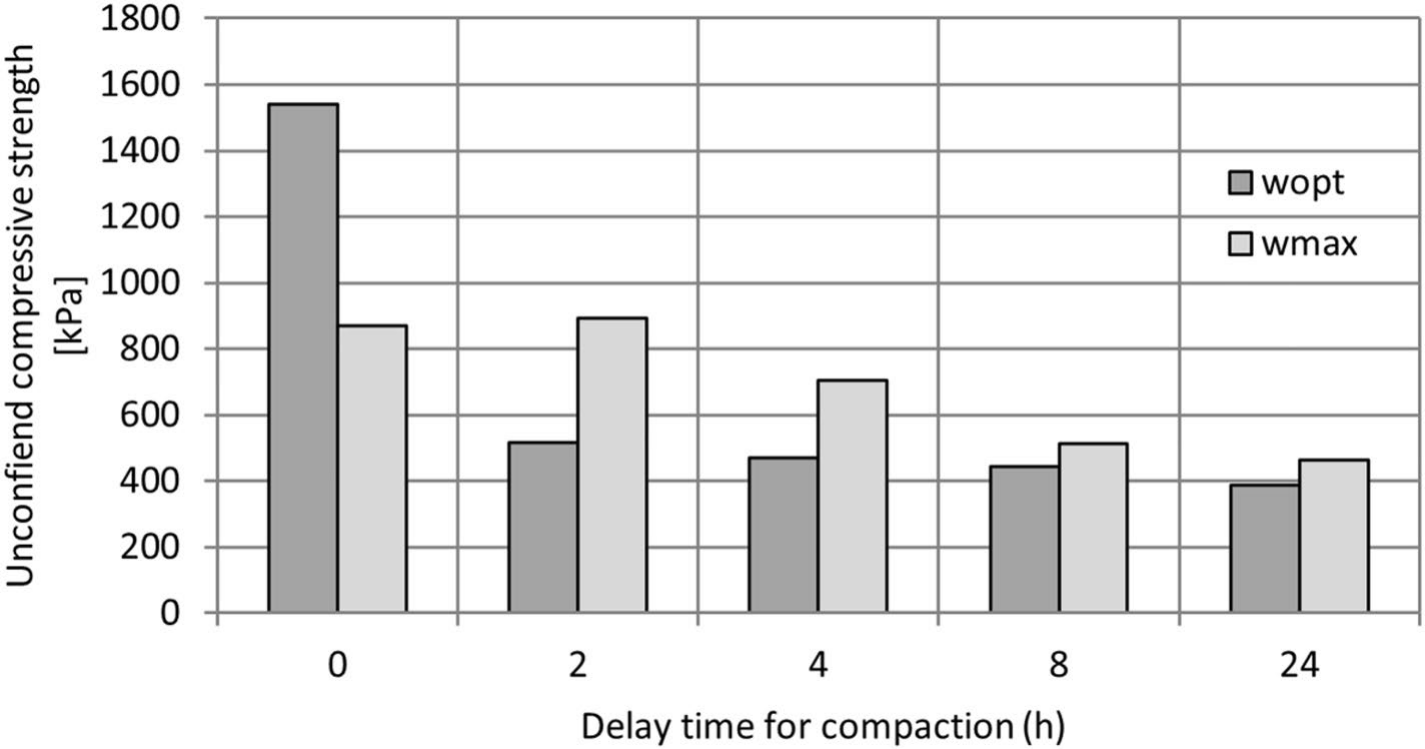
Unconfined compressive strength depending on the compaction time.

The geomechanical properties of the composite changed according to the time between mixing and compaction, but previous research has not put much emphasis on this parameter. DPSA is a hydraulically active material^31^. The results show that the uniaxial compressive strength decreases with increasing time between mixing and compaction (Fig. 8). The maximum value of the uniaxial compressive strength is reached if the material is compacted at w_opt_, immediately after mixing. If the material is compacted at w_max_, the uniaxial compressive strength is reduced by 40%. The situation is different when the material is compacted with a time delay. Even with a two-hour delay between mixing and compacting, the value of the uniaxial compressive strength decreases. In this case, the decrease is greater for mixtures compacted at w_opt_ (75%) and smaller for mixtures compacted at w_max_ (40%) in comparison with the highest value of uniaxial compressive strength. The hydration reaction causes drying of the material, which results in lower uniaxial compressive strength.

If the mixing and construction site is not at the same place, the transport time has a high impact on the uniaxial compressive strength. A decrease in the uniaxial compressive strength (11–37%) was reported with a compaction delay of 2 h for a mixture of expansive soil and cement^47^.

According to the results, the composite must be moistened to w_opt_, if the mixture was compacted immediately after mixing. If the mixture is to be transported to the construction site, it had to be moistened to w_max_ and compacted within 4 h. These results are very important to the optimisation of the mixture in dependence of the transport distance to the construction site. For the stability analysis of a retaining wall structure, the designer must have access to the detailed geomechanical parameters of the material at the time when it is installed in the structure, including the effects of the time lag between mixing and compaction.

#### Shear properties

**Figure 9:**
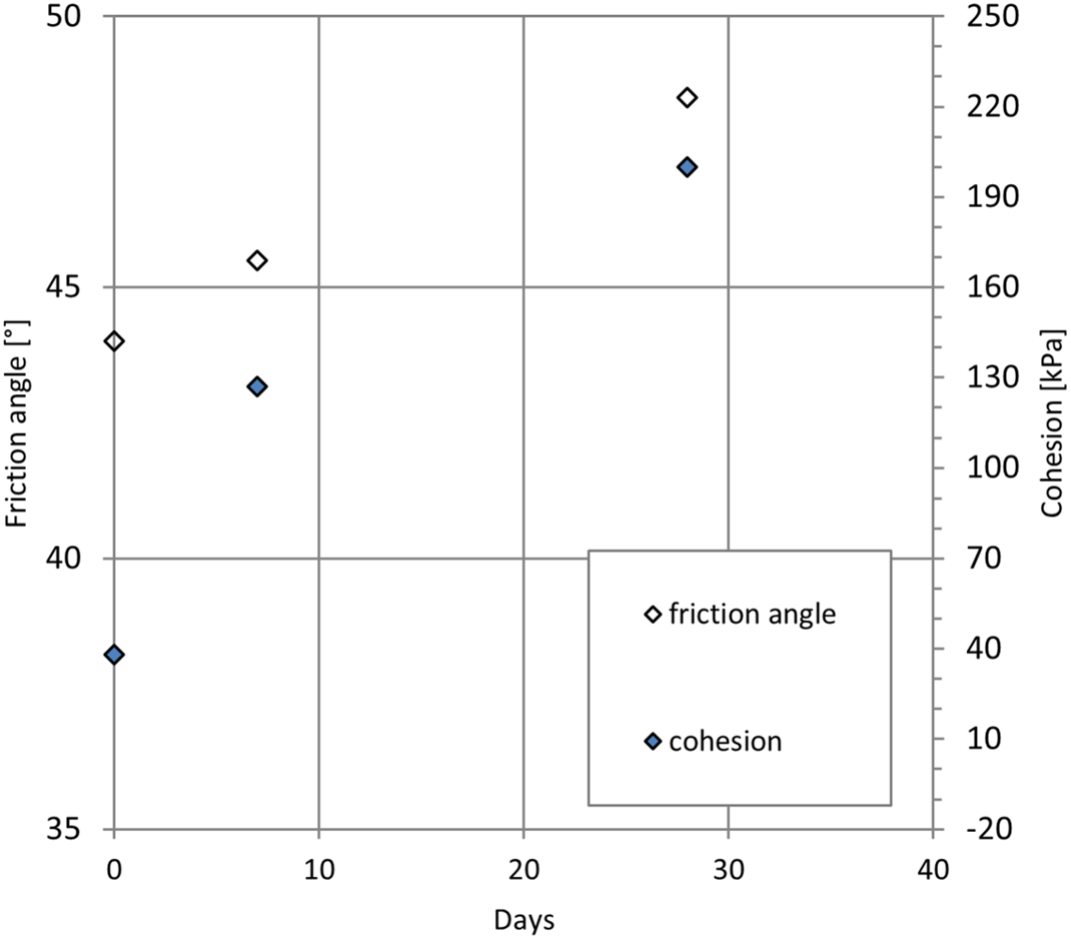
Friction angle and cohesion of the composite.

**Figure 10:**
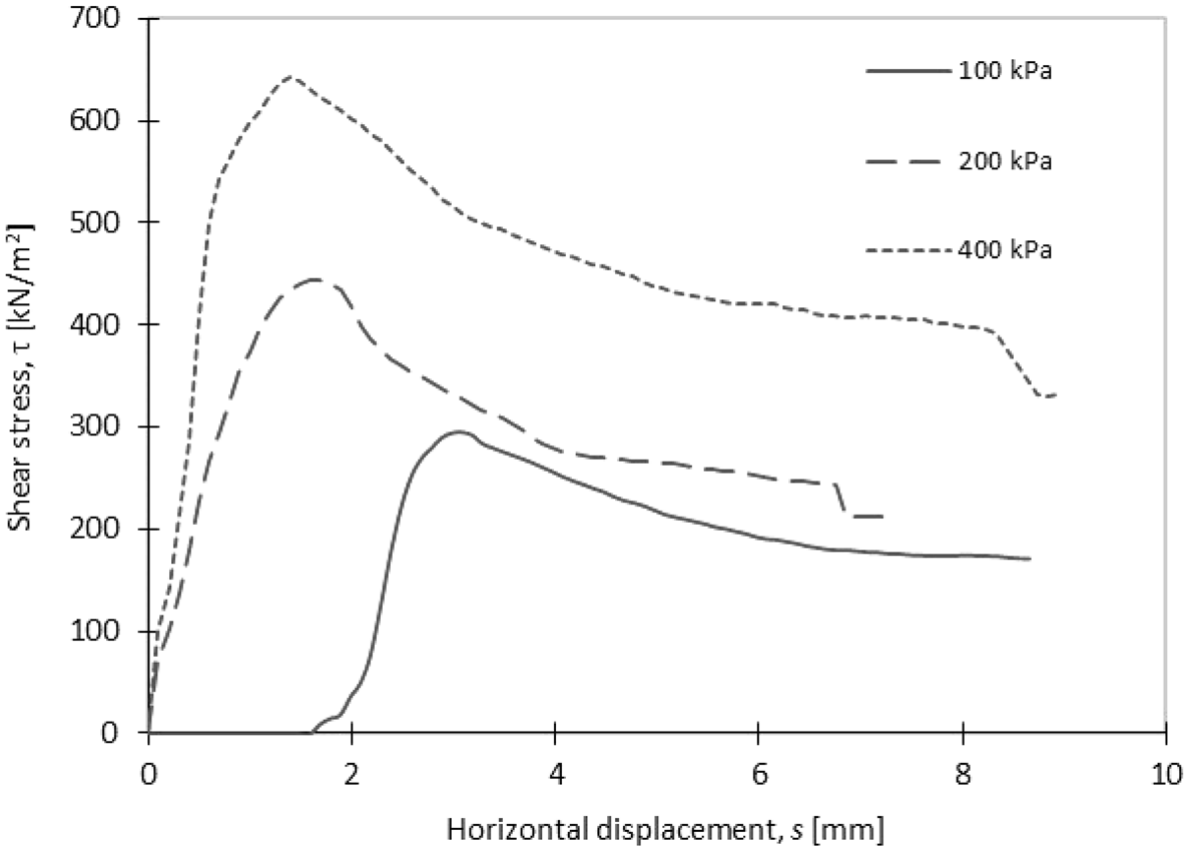
Shear stress vs horizontal displacement for the sample cured 28 days.

The results of the direct shear tests showed that the friction angle (∅′) and cohesion (c) increased with the time (Fig. 9). After 28 days of curing, the composite reached a friction angle of 48° and a cohesion of 200 kPa (Fig. 10). In comparison with the natural material, the shear properties of the composite were higher, i.e. gravel usually has a friction angle around 36° and no cohesion. A mixture of sand and 70% of fly ash reached lower values of friction angle (42°) and cohesion (84 kPa) even it included 3% of cement^48^.

#### Frost resistance

The samples were subjected to a frost investigation with 12 cycles of freezing and thawing. Freezing ration is coefficient between the unconfined compressive strength after exposure to freezing and the unconfined strength after sample curing at atmospheric temperature and humidity. According to the Slovenian legislation^41^ it must be more than 0.7. The results indicated that the composite could be used at temperatures below 0 °C because the freezing ratio was more than 0.7 (measured value 0.76).

Based on the laboratory results, mixture C70/30 was tested in the small demo site with the different time of compaction. The results from the laboratory were confirmed with the field observations. If the mixture is transported from the paper factory to the construction site, the moisture of the mixture has to be higher than w_opt_ , while 3 to 5% of water were consumed based on hydration processes in the composite. In the laboratory, q_u_ was lower for the mixture compacted 4 h after mixing, and the same result was obtained from the field test measurements.

### Results from leaching tests

Leaching tests were performed on samples taken from the small demo sites compacted with a lag of 4 h. It was proved that PSA is a hydraulically active material^31^. Hydrocalumite and monocarboaluminate were identified in composites after 50 days. Hydraulically processes in the composite have provided that all concentrations of elements were below the limits set for inert waste by the current Slovenian legislation^49^ Thus, the composite does not entail any negative environmental impacts.

### Results from the large demo retaining wall structure

Based on the laboratory results and those from the construction of small test sites, the decision of using composite D30/70 was confirmed. Prior to construction work, a large quality control was organised with field and laboratory testing.

#### Results of laboratory tests of samples from the large demo retaining wall

**Figure 11:**
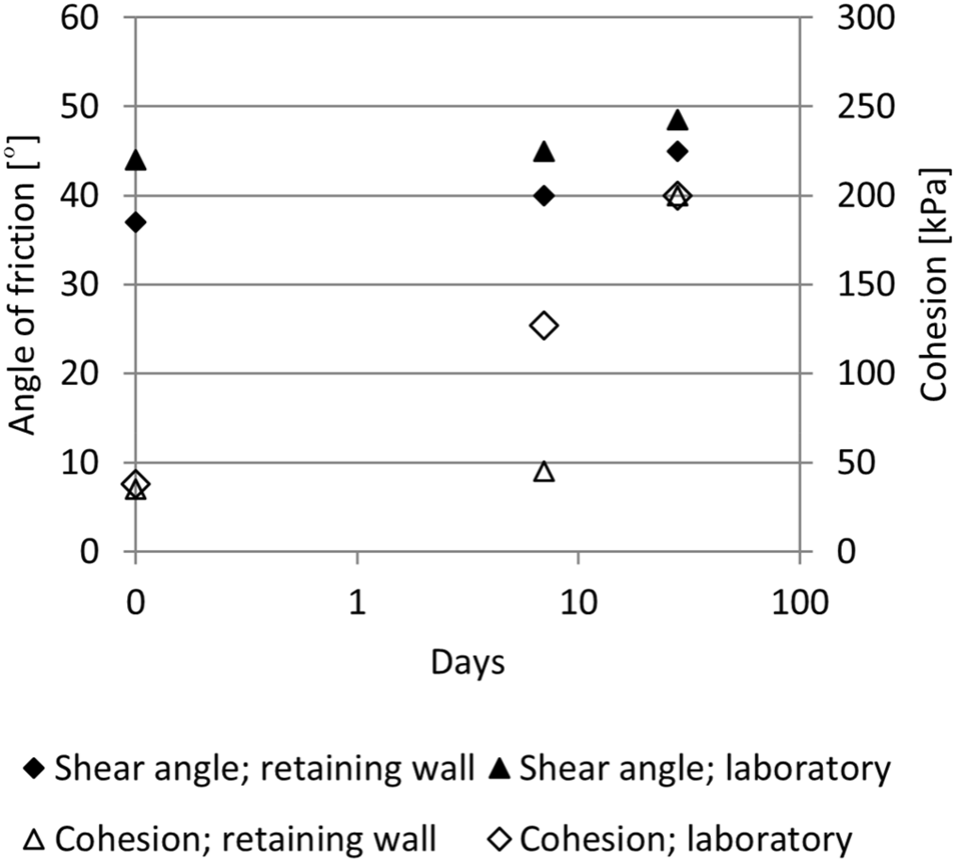
Shear properties from the samples prepared in the laboratory and samples from the structure.

Samples from the last layer of the composite were taken to the laboratory for the testing. The direct shear test was performed immediately after compaction and results showed that the angle of friction was lower by 15% in comparison with the previous laboratory tests in the first phase, when the laboratory prepared composite was tested. The cohesion was almost the same for both samples. After one week of curing the angle of friction and cohesion increased. However, there was still a difference between the samples taken from the structure and those tested in the laboratory, where the cohesion was much lower for the retaining wall. After 28 days, the results for both samples were very similar; only the angle of friction was higher by 10% for the samples prepared in the laboratory (Fig. 11). In the construction site, the composite could not be as homogenously compacted as in the laboratory, so that the shear parameters in the actual wall were slightly lower (angle of friction), while the results still matched very well. The shear characteristics of the composite are higher than the shear characteristics of the compacted gravel material.

In comparison with the original gravel material, the new composite has much better shear characteristics, especially cohesion, which is comparable to those of soft rock.

Leachate tests were performed on samples taken from the installed composite, which confirmed that the material has no negative environmental impacts. The composite did not exceed any limits for hazardous substances according to Slovenian legislation^49^.

#### Field tests

The percent of compaction was calculated based on nuclear probe measurements (SI1). Specification TSC 06.711 requires the compaction of the layers has to be at least 92% of ρ_d,max_. The measurement results showed that the installed layers reached the required limits.

The dynamic modulus has to reach 15 MPa according to the requirements of the Slovenian technical specification^50^. The results were like those from the small demo sites. After 7 days, all dynamic modulus values were higher than 15 MPa.

After the structure had been finished, the composite was covered with a humus layer. Nowadays, the composite is covered with field plants, and the whole construction fits very naturally into the environment.

#### Results of long-term monitoring

**Figure 12:**
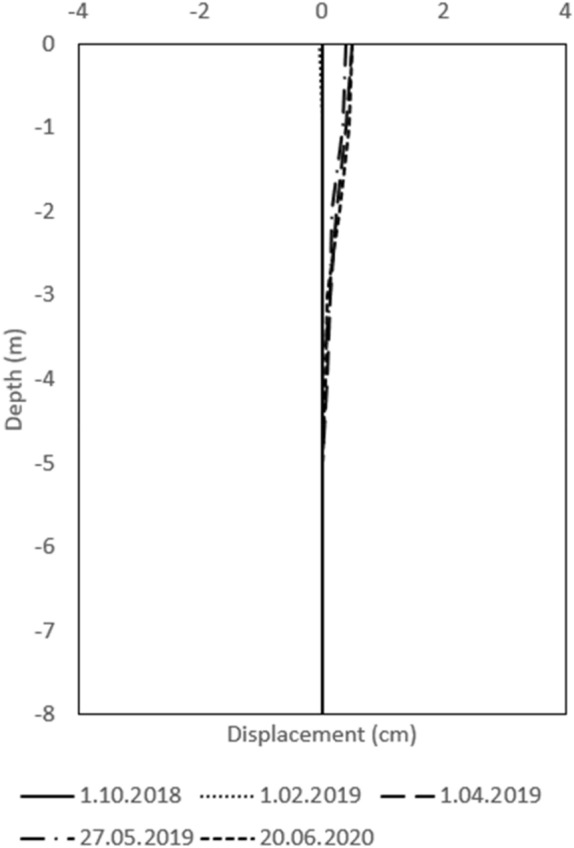
Inclinometer measurements in borehole P1.

**Figure 13:**
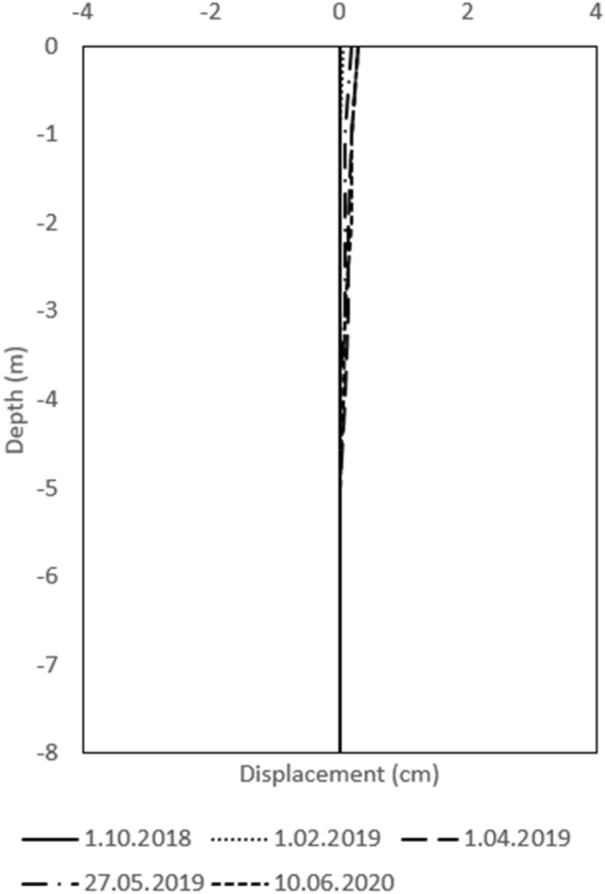
Inclinometer measurements in borehole P3.

To ensure the safety of the railway line from landslides, a retaining wall was built, which has now been observed for more than two years. The landslide stability is observed with two inclinometers (P1, P3) and horizontal displacements were acceptable (Figs. 12, 13). Water level measurements are also carried out in the inclinometer well. The location of the inclinometers is visible in the Fig. 1.

**Figure 14:**
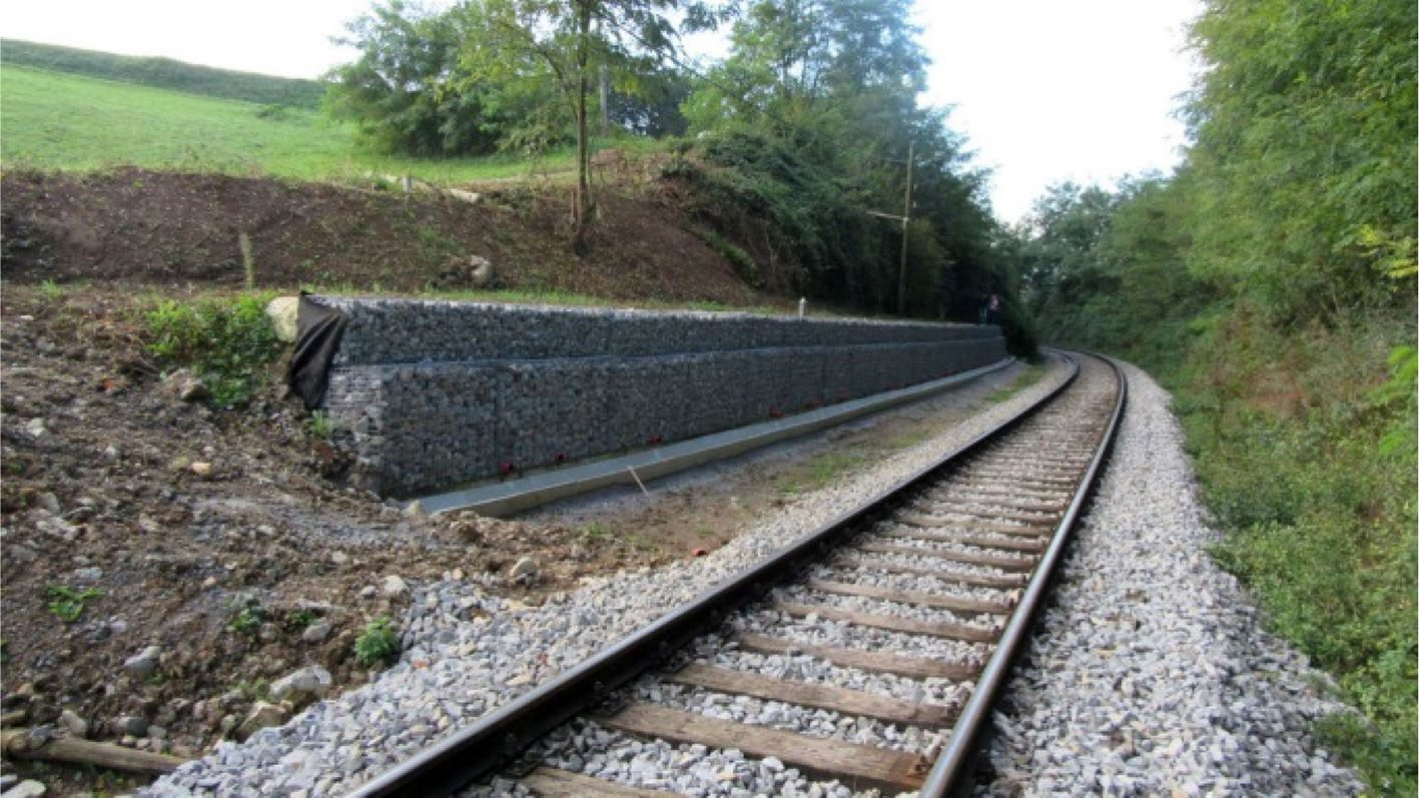
Picture of the retaining wall structure.

**Figure 15:**
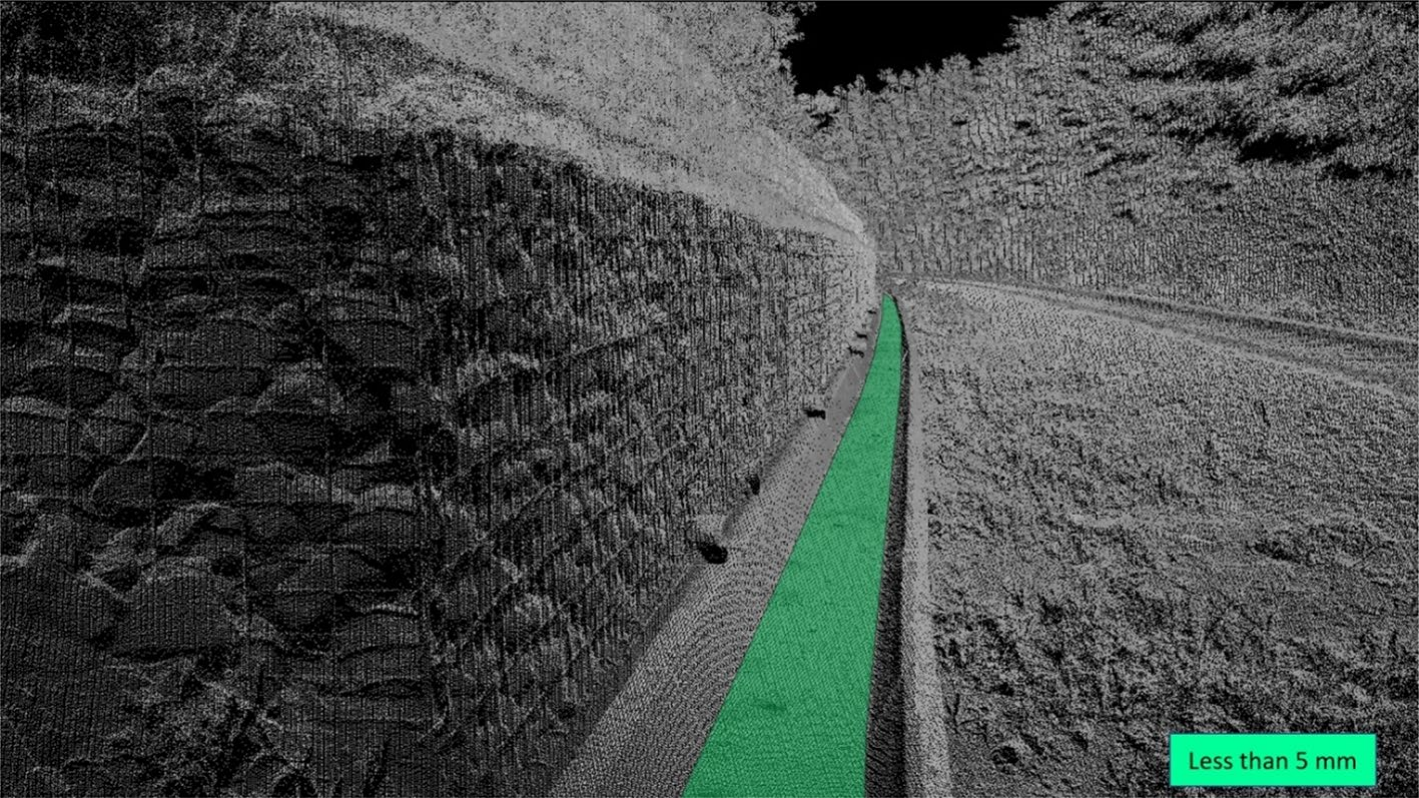
Scan of the retaining wall structure; the difference in deformations between 2019 and 2020.

The retaining wall stopped landslide movement successfully. The stability of the landslide was also measured using detailed laser scanner measurements. The difference between the measurements taken in 2019 and 2020 showed that that there were no displacements and cracks on the retaining wall structure and on the surface of the landslide (Figs. 14, 15). Deformations only happen in the drainage system where the settlements are less than 5 mm. In a time of measuring (year 2020) the concrete drainage canal next to the gabions has not yet been concreted.

Samples were taken from the last layer of the large demo retaining wall structure for estimation the environmental impact of the used composite. Leaching test was performed on the samples curing 28 days. The results showed (Table 4) that none of the components in the leachate exceeded the limits established by Slovenian legislation^49^. This also applies to barium (Ba) which is the only metal element above the limits of the inert waste in the waste material.

**Table 4:** Concentrations of elements in the leachate from DPSA and DPS.

Component		As	Ba	Cd	Cr total	Cu	Mo	Ni
Average	mg/kg d m	0.003	8.82	< 0.002	0.002	0.61	0.097	0.006
Limit	mg/kg d m	0.5	20	0.04	0.5	2	0,5	0.4

Component		Pb	Sb	Se	Zn	Chloride	Fluoride	Sulphates
Average	mg/kg d m	0.005	0.001	0.003	0.005	13.5	3.6	10
Limit	mg/kg d m	0.5	0.06	0.1	4	800	10	1000

Water samples collected from the water tank installed near the structure were taken and analysed in an accredited chemical laboratory. The result of the sample, taken almost 2 years after the structure was finished, is presented in Table 5 and results of chemical analysis of water for year 2019 and 2020 are presented in SI5. The tested parameters did not exceed the limits prescribed by Slovenian legislation^51^. It could be concluded that the composite meets all environmental requirements according to the national law.

**Table 5:** Chemical analysis of the water.

Component		As	Ba	Cd	Cr total	Cu	Mo	Ni
Average	mg/l	0.0014	0.046	< 0.0002	0.005	0.022	0.0077	0.0017
Limit	mg/l	0.1	5	0.025	0.5	0.5	1	0.5

Component		Pb	Sb	Se	Zn	Chloride	Fluoride	Sulphates
Average	mg/l	0.0006	0.0022	0.0004	0.0011	2.96	0.234	12
Limit	mg/l	0.5	0.3	0.6	2	800	10	1000

Results from installed probes for measuring temperature and for the water content showed that the composite has low water permeability coefficient while no of the precipitation have influence on water content of the backfill (SI2–SI4). This was also confirmed with the sample that was taken after one year from the upper backfill layer and was tested in the laboratory for the water content.

The monitoring of the retaining wall continues to confirm the results so far.

## Conclusions

The aim of this study was to develop a backfill material for geotechnical structures. In the paper the validation for the retaining wall structure is presented. For new composite the mixture from paper sludge ash and paper sludge from the residues of the paper industry in the ratio 70:30 was used. The retaining wall structure with the new composite was built near the railway line to prevent further movement of a landslide. The composite had a sufficient uniaxial compressive strength and shear properties and allowed small deformations before reaching the peak strength. The technology and procedure of material compaction were first tested in small demo sites, and finally, the large demo retaining structure was built. The described composite has not yet been used as a backfill material together with the gabions.

The laboratory results showed that the geomechanical properties of the composite decreased with the time between mixing and compaction. If PSA and PS are mixed at paper factory facilities, the time of transport to the construction site is very important, to which previous research has, to date, not paid attention to. The results from the laboratory and small demo sites showed that before transport, the composite had to be moistened above w_opt_. This is very important statement which has to be considered when the backfill material is used. If the mixture has not sufficient water content, the dynamic elastic modulus of the compacted layer would be lower as required by legislation. Three to 5% of water was consumed based on the hydration reactions of PSA. Small demo sites were prepared immediately after compaction and with a lag of 4 and 24 h. Similar to the laboratory tests, the results showed that the composite could be properly compacted even 4 h after mixing.

In 2019, the construction of the large demo structure was finished in the southern part of Slovenia. For the retaining wall structure, gabions and backfill material based on the presented composite were used. An intense quality control of geomechanical properties was conducted during the construction phase. Every layer was tested according to density, water content, and dynamic modulus. Several samples were taken to a geomechanical laboratory to confirm the geomechanical properties and environmental parameters. The results confirmed that the geomechanical properties of the installed composite were similar to those determined in the preliminary laboratory tests. Leaching tests of the used composite showed that material does not have any environmental impact. Thus, the composite meets all technical and environmental requirements required for backfill material.

A two-year monitoring after construction confirmed the stability of the landslide and the environmental acceptability of the new composite. The monitoring of the retaining wall continues to confirm the results so far.

The new composite has proven to promote the advantages of a circular economy. The composite meets the same technical and environmental requirements as the natural backfill material. The large demo structure is an example of the knowledge transfer from the laboratory to the construction site, in which large demo structures are crucial for testing composite and installing technology. Via installation control and the monitoring of geomechanical and environmental parameters, it was proven that the new composite could meet all sustainable requirements and is, with a suitable economic model, also cheaper for the contractor than the natural materials. The successfully built large demo retaining wall structure with the new backfill composite could encourage suppliers, designers, construction companies, and investors to replace the natural material with the recycled one. It may serve as a role model for a material cycle between the paper and construction industry.

### Supplementary Information

Supplementary Information.

## Data Availability

The datasets used and/or analysed during the current study available from the corresponding author on reasonable request.
