# Supplementary material for: Validation of the recycled backfill material for the landslide stabilization at a railway line
Source: Sci Rep. 2024 Mar 25;14:7031. doi: 10.1038/s41598-024-57555-4 (PMC10963361; doi:10.1038/s41598-024-57555-4)
Supplement: Supplementary file 1 — Supplementary Information. [file 41598_2024_57555_MOESM1_ESM.docx]

SUPPLEMENTARY INFORMATION

Supplementary Figures

Figure SI1 Compaction of the installed layers in the structure

Figure SI 2 Precipitation and water content of the composite layer

Figure SI3 Temperature changes during year 2021

Figure SI4 Temperature during year 2021 - 2022

Table SI5 Chemical analysis of the water in year 2019 and 2020

| **Component** | **Limit** | **Water sample**  **April 2019** | Water sample  May 2019 | Water sample  October 2019 | Water sample  June 2020 | Water sample  November 2020 |
| --- | --- | --- | --- | --- | --- | --- |
|  | **(mg/l)** | | | |  |  |
| **As** | **0.1** | **0.0019** | 0.0006 | 0.0017 | 0,0019 | 0,0007 |
| Ba | 5 | 0.0080 | 0.0073 | 0.122 | 0,04 | 0,063 |
| Cd | 0.025 | < 0.0002 | < 0.0002 | < 0.0002 | < 0,0002 | < 0,0002 |
| Cr total | 0.5 | 0.0031 | 0.0010 | 0.011 | 0,0015 | 0,0009 |
| Cu | 0.5 | 0.042 | 0.011 | 0.013 | 0,039 | 0,031 |
| Hg | 0.005 | < 0.0001 | < 0.0001 | < 0.0001 | < 0,0001 | < 0,0001 |
| Mo | 1 | 0.018 | 0.0024 | 0.0028 | 0,028 | 0,02 |
| Ni | 0.5 | 0.0011 | 0.0015 | 0.0024 | 0,003 | 0,0015 |
| Pb | 0.5 | 0.0005 | 0.0006 | < 0.0005 | < 0,0005 | < 0,0005 |
| Sb | 0.3 | 0.0039 | 0.0011 | 0.0017 | 0,0036 | 0,0034 |
| Se | 0.6 | 0.0005 | < 0.0003 | 0.0003 | 0,0003 | < 0,0003 |
| Zn | 2 | < 0.0005 | 0.0016 | 0.0005 | 0,0015 | 0,011 |
| Chlorides | 800 | 5.17 | 1.52 | 2.19 | 11 | 8 |
| Fluorides | 10 | 0.26 | < 0.10 | 0.20 | 0,28 | 0,35 |
| Sulphates | 1000 | 19 | 2 | 14 | 20 | 20 |
